# Comparative Study of Emotional Intelligence and Perceived Stress Among Dental Postgraduate Students

**DOI:** 10.7759/cureus.79392

**Published:** 2025-02-21

**Authors:** Vian M Hussein

**Affiliations:** 1 Dental Public Health, Kurdistan Higher Council of Medical Specialties, Erbil, IRQ

**Keywords:** dental education, emotional intelligence, kurdistan region, perceived stress, postgraduate students

## Abstract

Introduction: Dental education is a vigorous and stressful process characterized by academic, clinical, and patient care demands. Research suggests better stress management is associated with emotional intelligence (EI), the capacity to appreciate and control emotions. The aim of this study is to evaluate EI and perceived stress levels among dental postgraduate students in the Kurdistan region and examine their correlation as well as identify key influencing factors.

Methods: A cross-sectional study that included 190 postgraduate dental students from Erbil, Sulaymaniyah, and Duhok was conducted between March and July of 2024. The perceived stress scale (PSS) evaluated stress levels, and the Schutte Self-Report Emotional Intelligence Test (SSEIT) evaluated EI through emotion appraisal, regulation, and utilization.

Results: 38.6% of the respondents reported high levels of stress. There was a statistically significant difference between the EI scores of females and males (p = 0.045), with female students having higher EI scores than males. Lower stress and higher EI scores were observed in first-year students as compared to third-year students. While higher EI was associated with less stress (weak negative correlation, r = -0.237, p > 0.05), this association was not statistically significant in this study population, suggesting that while high EI may assist in regulating stress, the observed effect shown was non-significant.

Conclusion: The current study confirmed the high level of stress among dental students in the Kurdistan region and the effectiveness of EI in improving it. The results of our study suggest that the incorporation of EI training in dental curricula, especially for students close to graduation, would help to improve resilience and mental health. Structured EI workshops and stress management interventions tailored to the specific needs of students may enable students to effectively manage academic and clinical stressors.

## Introduction

Dental education imposes important academic and clinical challenges for students, which leads to a high level of stress [1]. These pressures stem from the need to learn technical skills, care for patients, and perform well academically. This creates an environment where mental health issues and disorders thrive, including anxiety and burnout [2]. Even though many studies have reported stress levels in dental students worldwide, the focus on dental students in the Kurdistan region was very limited. This gap demonstrates a need for research that is appropriate for this specific population, given its unique socio-cultural and academic contexts.

Emotional intelligence (EI) is the capacity to identify, comprehend, and regulate one’s own emotions and those of others and has been identified as an important factor in managing stress [3]. In particular, trait EI is linked with better stress management, coping, and psychological functioning [4]. In health education, especially in the field of dentistry, high EI enables students to meet the emotional challenges associated with caring for patients [5].

Studies investigating the association between EI and perceived stress scale (PSS) among healthcare students have shown that the PSS levels were lower in individuals with higher EI [6]. Guerra-Bustamante(2019) found that EI predicts psychological well-being through adaptive coping strategies, while Jiménez-Picón et al. (2021) highlighted its role in managing stress among healthcare professionals [7, 8]. However, research regarding EI and stress regulation by gender is not fully consistent in the literature, and much research has been focused on Western populations using relatively small samples [9]. The methodological restraints inhibit the generalizability of the findings to culturally heterogeneous settings, such as the Kurdistan region. In addition, the use of self-reported measures of stress without controlling for confounding factors such as academic workload and cultural differences highlights the necessity for a more nuanced approach [10].

To fill these gaps, this study relies on Mayer and Salovey’s EI model based on the assumption that people with high EI can identify and manage stressors [11]. However, EI is believed to be positively correlated with stress response and regulation, which helps inform the theoretical basis of our study. Christopher and Staci (2020) verify that our literature review is consistent with the latest trends and research made in the field [10,12].

This study aims to assess the relationship between EI and perceived stress among postgraduate dental students at the Kurdistan Higher Council for Medical Specialties (KHCMS). An additional goal is to uncover possible areas for intervention in order to improve student well-being and academic success. We hypothesized that greater EI would be associated with lower perceived stress, in line with theoretical models suggesting that EI is a mechanism for promoting constructive coping styles and good mental health [13].

Besides filling the research gap in the Kurdistan region, this study also aims to contribute to the design of EI training programs in dental education. These initiatives would help build capacity and mental health for dental students, which would further allow them to thrive in their profession.

## Materials and methods

Study design and participants

This study was conducted as a cross-sectional survey among 190 postgraduate dental students enrolled in the specialty board programs under the Kurdistan Higher Council of Medical Specialties in Erbil, Sulaymaniyah, and Duhok in the Kurdistan region of Iraq. The specialty board in the Kurdistan Region of Iraq is a structured five-year training program, meaning that students in this study were in different years of their postgraduate education, ranging from year one to year five. This study focused only on postgraduate dental students because their high academic level and clinical experiences are more suited to the study’s purposes.

Given the limited availability of postgraduate students (who have both clinical and academic commitments), we used convenience sampling, which is known to be more efficient but has also undoubtedly introduced bias. Although stratified random sampling was an option, it was not feasible considering the institutional arrangement. Although this method restricts generalizability, we discuss this limitation in the discussion section.

The sample size was calculated using GPower software for a significance level = 0.05, power = 80%, and expected medium effect size (Cohen’s d = 0.5), resulting in a minimum necessary sample size of 85 participants per group. The number of subjects was increased to 190 to compensate for possible non-responders.

Data collection

An explicit English version of the questionnaire was launched, and each participant had already been judged to be capable of English for completion in their postgraduate programs.

Pilot study

To assess the clarity of the questionnaire, a pilot study with 15 postgraduate students was carried out. In feedback, all items were reported as understood, with minor changes made to wording for increased precision, and required no structural changes to make it work. The Cronbach’s alpha of the questionnaire was confirmed to be 0.82 for PSS and 0.87 for the Schutte Self-Report Emotional Intelligence Test (SSEIT), indicating their internal consistency.

Data were collected using the PSS and the SSEIT. Both tools were validated instruments with known reliability, which have been practiced in comparable research. PSS scores were grouped into low (0-19), moderate (20), and high (>20) stress levels (Table 1). The SSEIT measured emotional intelligence on three dimensions: appraisal and expression of emotions, regulation of emotions, and utilization of emotions (Table 2).

**Table 1:** Summary of Perceived Stress Scale (PSS) Scores Credits: Cohen et al. [14]. Permission to use the Perceived Stress Scale was obtained from the original publisher. The scale is reprinted with permission from the American Sociological Association.

Category	Range of Scores	Description
Low Stress	0–19	Minimal perceived stress
Moderate Stress	20	Moderate perceived stress
High Stress	>20	High level of stress

**Table 2:** Summary of Emotional Intelligence (EI) Components Based on SSEIT Credits: The Schutte Self-Report Emotional Intelligence Test (SSEIT) was developed by Schutte et al. [15]. The instrument is included in this study with proper attribution to the original authors.

EI Component	Number of Items	Focus
Appraisal and Expression of Emotions	13	Identifies how individuals evaluate and express emotions
Regulation of Emotions	10	Measures emotional regulation strategies
Utilization of Emotions	10	Assesses how emotions are applied to problem-solving tasks

Adjusting for confounders

We used certain inclusion and exclusion criteria in order to avoid the influence of possible confounding factors. Only students who voluntarily signed up for and answered the questionnaire were counted. The analysis did not directly measure but considered these potentially confounding factors, such as academic workload, personal circumstances, and existing mental health conditions, that may influence stress and EI levels. For example, the sample was stratified by academic specialty and gender to detect differences in stress and EI within certain subgroups. More in-depth measures of such confounding variables would be beneficial in future studies.

Exclusion criteria

Dental students who did not agree to take part in the study were among the exclusion criteria.

Inclusion criteria

Eligible participants were postgraduate dental students who provided written consent to participate and answered all items of the questionnaires.

Data management and statistical analyses

Data were obtained through a specially designed questionnaire, which was reported and inputted into Microsoft Excel (2016). The finally gathered data were statistically analyzed by using Statistical Package for Social Sciences (SPSS) software version 28, and the level of significance was set at ≤ 0.05. Results were reported as rates, ratios, frequencies, and percentages and analyzed using a t-test and Chi-square test.

Ethical considerations

The study was approved by the Ethics and Scientific Committees of the Kurdistan Higher Council of Medical Specialties (Ethical code number: 1362-15/5/2024). All participants were informed about the purpose of the research, and verbal consent was obtained. Strict data confidentiality and anonymity were guaranteed.

## Results

This study included a total of 190 participants, of whom more than half (53.2%) were females and 46.8% were males. Mean age ± standard deviation (SD) of participants: 33.58 ± 3.29 years. The mean PSS was 19.39 ± 5.212, and the EI mean was 115.18 ± 19.942. These results are summarized in Table 3.

**Table 3:** Characteristics of the study participants (N = 190)

Variable	N	Range (Min-Max)	Mean ± SD
Age (years)	190	27–42	33.58 ± 3.290
Perceived Stress Scale	190	10–33	19.39 ± 5.212
Emotional Intelligence	190	37–151	115.18 ± 19.942

Relationship between sex and PSS and EI

Table 4 presents the relationship between sex and PSS and EI. There was no significant association between sex and PSS using the chi-square test (p = 0.566). However, a weak significant association was detected between the sex and EI (p = 0.045). In females, the results were 74 (73.3%) high EI and 53 (59.6%) males. At the very low EI levels, 36 (40.4%) were males, and only 27 (26.7%) were females.

**Table 4:** Association between sex, perceived stress scale (PSS), and emotional intelligence (EI)

Variable	Categories	Male N (%)	Female N (%)	Chi-square	p-value
PSS	Low stress	46 (51.7%)	54 (53.5%)	1.142	0.566
	Moderate stress	9 (10.1%)	6 (5.9%)		
	High stress	34 (38.2%)	41 (40.6%)		
EI	Low EI	36 (40.4%)	27 (26.7%)	4.040	0.045
	High EI	53 (59.6%)	74 (73.3%)		

The association between grade and PSS and EI

The association between academic grade and PSS and EI is presented in Table 5. There was a significant association between grade and both PSS (p = 0.004) and EI (p = 0.037). Such as, 9 (75%) of non-EI first-year students had high EI while only 15 (45.5%) of the other EI second-year students were in the high EI category. In addition, 23 (34.8%) of fifth-year students had high stress, compared with 3 (25%) of first-year students.

**Table 5:** Association between academic grade and PSS and EI PSS: Perceived Stress Scale; EI: emotional intelligence

Variable	Categories	1st Year	2nd Year	3rd Year	4th Year	5th Year	Chi-square	p-value
PSS	Low stress	9 (75%)	21 (63.6%)	13 (38.2%)	26 (57.8%)	31 (47%)	15.345	0.004
	Moderate stress	0 (0%)	0 (0%)	3 (8.8%)	0 (0%)	12 (18.2%)		
	High stress	3 (25%)	12 (36.4%)	18 (52.9%)	19 (42.2%)	23 (34.8%)		
EI	Low EI	3 (25%)	18 (54.5%)	8 (23.5%)	11 (24.4%)	23 (34.8%)	9.105	0.037
	High EI	9 (75%)	15 (45.5%)	26 (76.5%)	34 (75.6%)	43 (65.2%)		

Trends in stress and emotional intelligence

**Figure 1:**
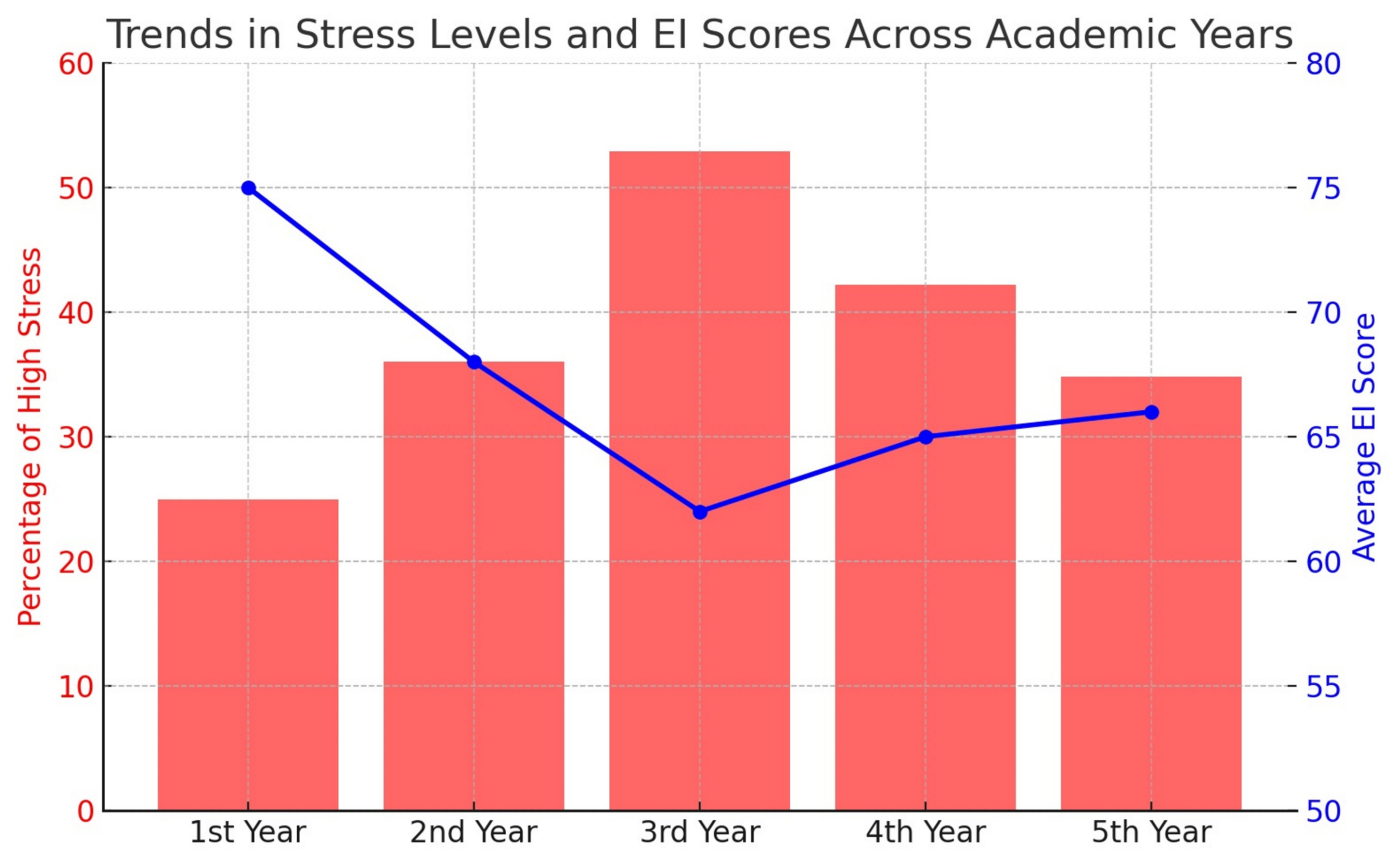
Prevalence of stress levels and emotional intelligence (EI) scores among postgraduate dental students in various academic years 🔴 Red Bars: Percentage of high stress students (using Perceived Stress Scale (PSS)) 🔵 Blue Line shows average EI scores (using the Schutte Self-Report Emotional Intelligence Test (SSEIT)). The image is created by the author of this article.

The results of this study show increasing levels of stress as students continue through postgraduate training, where third-year students experienced the highest levels of stress (52.9%), and first-year students revealed the lowest levels (25%). In contrast, EI scores tend to go down during training as students get further into their program. This trend is exemplified in Figure 1, which displays stress levels* and EI scores across academic years.

Relationships between age, specialties, and PSS and EI

The correlation between EI and PSS showed a weak (negative correlation of EI and PSS (r = -0.044, p = 0.544)) and non-significant. There was no significant statistical association between EI or PSS levels and age groups (p > 0.05, Table 6). There was likewise no significant association between specialties (dental public health versus other specialties) with either PSS and EI (p > 0.05, Table 7).

**Table 6:** Association between age and EI/PSS PSS: Perceived Stress Scale; EI: emotional intelligence

Variable	Categories	Age (Mean ± SD)	p-value	Statistical Test	Pearson Correlation	Sig. (2-tailed)
EI	Low EI	33.32 ± 3.277	0.442	t-test	-0.044	0.544
	High EI	33.71 ± 3.303				
PSS	Low stress	33.41 ± 3.537	0.082	ANOVA		
	Moderate stress	35.40 ± 4.120				
	High stress	33.44 ± 2.642				

**Table 7:** Association between specialties and PSS/EI PSS: Perceived Stress Scale; EI: emotional intelligence

Variable	Categories	Other Specialties N (%)	Dental Public Health N (%)	Chi-square	p-value
PSS	Low stress	88 (54.7%)	12 (41.4%)	1.541	0.416
	Moderate stress	12 (7.5%)	3 (10.3%)		
	High stress	61 (37.9%)	14 (48.3%)		
EI	Low EI	51 (31.7%)	12 (41.4%)	1.040	0.307
	High EI	110 (68.3%)	17 (58.6%)		

## Discussion

This study aimed to explore the relationship between EI and PSS among postgraduate dental students of the Kurdistan Higher Council for Medical Specialties (KHCMS). Dental education naturally involves significant stress stemming from academic demands, clinical work, and the challenges of managing patients. Understanding how EI influences stress levels can provide valuable insights into improving the mental health and academic performance of dental students.

The study found that 39.5% of respondents reported experiencing high stress levels, aligning with international studies that highlight the substantial stress commonly faced by dental students during their training. Previous studies have reported similar stress prevalence among dental students due to the unique demands of dental education, which include mastering technical skills, dealing with patient anxiety, and coping with rigorous academic schedules [16,17]. However, the specific context of dental students in the Kurdistan region had not been previously examined, making this study a significant contribution to the existing body of knowledge.

Our study found no significant association between gender and PSS, which was not in line with some previous research that indicated that stress levels in dental students were higher in females [17,18]. However, the findings showed a significant relationship between gender and EI, with female students displaying higher levels of EI compared to their male counterparts. This result aligns with earlier studies suggesting that women often score higher on EI measures [19], The higher EI among female students might indicate greater emotional adaptability, which could influence their ability to manage stress, other studies showed the reverse [20]. And some studies showed no difference in both genders [21].

When analyzing the relationship between EI and PSS, the findings indicated a slight negative relationship between EI and stress, suggesting that higher levels of EI are linked to marginally reduced stress, though the correlation lacked statistical significance. This finding contrasts with other studies where significant negative correlations between EI and stress have been observed, suggesting that EI can serve as a buffer against stress in healthcare students [4,22]. The discrepancy may be attributed to cultural and educational differences specific to the Kurdistan region or variations in measurement tools.

Notably, students’ academic grade levels demonstrated significant correlations with both perceived stress and EI. First-year students reported the lowest levels of stress, while third-year students experienced the highest. This pattern likely reflects the growing challenges of dental education as students advance, with third-year students typically facing more demanding clinical tasks, this was in line with a study done in Sri Lanka at the University of Colombo in which higher EI was associated with better academic performance especially in the final grade levels and at the same time associated with low stress level, and also self-satisfaction was associated with a high level of EI [23]. Additionally, first- and second-year students exhibited higher EI compared to their senior peers, indicating that EI may decline as stress intensifies during the course of dental training. This inverse relationship highlights the potential need for early and sustained EI training in dental curricula [24].

Employing validated instruments like the PSS and the SSEIT enhances the reliability of this research. However, the convenience sampling method and the cross-sectional design limit the ability to generalize findings or infer causality. Conducting longitudinal studies could offer more comprehensive insights into the progression of EI and stress levels throughout dental education.

Clinically, the findings highlight the critical need to incorporate EI training within dental education curricula. Initiatives aimed at improving EI could involve training sessions focused on emotional regulation, managing stress, and developing interpersonal abilities. Yadav et al. (2020) and Ranasinghe et al. (2017) have demonstrated that such interventions can improve EI and reduce stress among students [25, 26]. These initiatives could be particularly beneficial for students in their later years of training, where stress levels are highest.

Although this study has provided some useful findings, it is not without some limitations that need to be noted. The generalizability of the findings to other student populations may be limited due to the convenience sampling approach. Also, the cross-sectional design restricts the possibility of establishing a cause-effect relationship between EI and perceived stress. Other individual or environmental factors that are able to affect stress levels and EI were also not considered and could have affected the results. Longitudinal studies conducted in the future are recommended to ensure a deeper understanding of the evolution of stress and EI during the course of dental education.

Future research directions

To extend the present findings, future studies should investigate the following: Longitudinal studies measured their EI and stress levels during their dental education through some criteria; reducing anxiety and enhancing academic performance through EI interventions; investigating the impact of cultural norms and institutional frameworks on EI and stress reactions.

## Conclusions

This study offers an initial perspective on the association between EI and stress among postgraduate dental students in the Kurdistan region. Though the relationship was weak and not statistically significant, the data supports the role of EI as a potential buffer against stress. To better support students' mental health and academic success, this information emphasizes the importance of integrating EI training into dental education curricula and addressing cultural and institutional factors within dental education programs. Further studies are encouraged toward these dynamics in order to provide evidence-based mechanisms for exploring stress in dental education.

## Appendices

**Table 8:** The Schutte Self Report Emotional Intelligence Test (SSEIT) 1 = Strongly disagree   2 = Disagree   3 = Neither disagree nor agree   4 = Agree   5 = Strongly agree The Schutte Self-Report Emotional Intelligence Test (SSEIT) was developed by Schutte et al. [13]. This instrument is used in this study with proper attribution to the original authors.

Question					
1. I know when to speak about my personal problems to others.	1	2	3	4	5
2. When I am faced with obstacles, I remember times I faced similar obstacles and overcame them.	1	2	3	4	5
3. I expect that I will do well on most things I try.	1	2	3	4	5
4. Other people find it easy to confide in me.	1	2	3	4	5
5. I find it hard to understand the non-verbal messages of other people*	1	2	3	4	5
6. Some of the major events of my life have led me to re-evaluate what is important and not important.	1	2	3	4	5
7. When my mood changes, I see new possibilities.	1	2	3	4	5
8. Emotions are one of the things that make my life worth living.	1	2	3	4	5
9. I am aware of my emotions as I experience them.	1	2	3	4	5
10. I expect good things to happen.	1	2	3	4	5
11. I like to share my emotions with others.	1	2	3	4	5
12. When I experience a positive emotion, I know how to make it last.	1	2	3	4	5
13. I arrange events others enjoy.	1	2	3	4	5
14. I seek out activities that make me happy.	1	2	3	4	5
15. I am aware of the non-verbal messages I send to others.	1	2	3	4	5
16. I present myself in a way that makes a good impression on others.	1	2	3	4	5
17. When I am in a positive mood, solving problems is easy for me.	1	2	3	4	5
18. By looking at their facial expressions, I recognize the emotions people are experiencing.	1	2	3	4	5
19. I know why my emotions change.	1	2	3	4	5
20. When I am in a positive mood, I am able to come up with new ideas.	1	2	3	4	5
21. I have control over my emotions.	1	2	3	4	5
22. I easily recognize my emotions as I experience them.	1	2	3	4	5
23. I motivate myself by imagining a good outcome to tasks I take on.	1	2	3	4	5
24. I compliment others when they have done something well.	1	2	3	4	5
25. I am aware of the non-verbal messages other people send.	1	2	3	4	5
26. When another person tells me about an important event in his or her life, I almost feel as though I have experienced this event myself.	1	2	3	4	5
27. When I feel a change in emotions, I tend to come up with new ideas.	1	2	3	4	5
28. When I am faced with a challenge, I give up because I believe I will fail*	1	2	3	4	5
29. I know what other people are feeling just by looking at them.	1	2	3	4	5
30. I help other people feel better when they are down.	1	2	3	4	5
31. I use good moods to help myself keep trying in the face of obstacles.	1	2	3	4	5
32. I can tell how people are feeling by listening to the tone of their voice.	1	2	3	4	5
33. It is difficult for me to understand why people feel the way they do*	1	2	3	4	5

**Table 9:** Perceived Stress Scale (PSS) The Perceived Stress Scale (PSS) measures the degree to which situations in an individual's life are appraised as stressful. The questions are rated on a 5-point Likert scale ranging from 0 (Never) to 4 (Very Often). The Perceived Stress Scale (PSS) was developed by Cohen et al. [12]. Permission to use and reproduce this scale was explicitly obtained from the original publisher. The scale is reprinted here with the consent of the American Sociological Association. This questionnaire is not published under a Creative Commons License.

Question Number	Question	Response Options (0 = Never, 1 = Almost Never, 2 = Sometimes, 3 = Fairly Often, 4 = Very Often)				
1	In the last month, how often have you been upset because of something that happened unexpectedly?	0	1	2	3	4
2	In the last month, how often have you felt that you were unable to control the important things in your life?	0	1	2	3	4
3	In the last month, how often have you felt nervous and stressed?	0	1	2	3	4
4	In the last month, how often have you felt confident about your ability to handle your personal problems?	0	1	2	3	4
5	In the last month, how often have you felt that things were going your way?	0	1	2	3	4
6	In the last month, how often have you found that you could not cope with all the things that you had to do?	0	1	2	3	4
7	In the last month, how often have you been able to control irritations in your life?	0	1	2	3	4
8	In the last month, how often have you felt that you were on top of things?	0	1	2	3	4
9	In the last month, how often have you been angered because of things that were outside of your control?	0	1	2	3	4
10	In the last month, how often have you felt difficulties were piling up so high that you could not overcome them?	0	1	2	3	4
